# JAK2-STAT5 signaling is insensitive to porcine growth hormone (pGH) in hepatocytes of neonatal pig

**DOI:** 10.1080/19768354.2020.1735518

**Published:** 2020-04-27

**Authors:** Yang Yu-Jiang, Zheng Xin, Lan Hai-Nan

**Affiliations:** College of Animal Science and Technology, Jilin Agricultural University, Changchun, People’s Republic of China

**Keywords:** Porcine growth hormone, growth hormone receptor, JAK2-STAT5/3/1, neonatal pig, porcine hepatocytes

## Abstract

Porcine growth hormone (pGH) is most important hormone which is involved in the growth and development of pig. However, a series of studies have indicated that neonatal pig is insensitive to pGH; the reason for this phenomenon is still not fully understood. In this work, we try to investigate this issue from the angle of intracellular signaling induced by pGH. In the present study, porcine hepatocytes from neonatal pig were used as a model, and confocal laser scanning microscopy (CLSM), Western blot, co-immunoprecipitation and colocalization assay were used to study pGH’s signaling properties in hepatocytes of neonatal pig and explore the possible mechanism(s) for why intracellular signaling is insensitive to pGH. The results indicated that Janus kinase 2 and signal transducers and activators of transcription 5/3/1 (JAK2-STATs) signaling are not activated. We further investigated the possible mechanism(s) by which JAK2-STATs’ signaling is not activated by pGH and growth hormone receptor (GHR) and found that the negative regulatory molecules of JAK2-STATs signaling may be associated with this phenomenon in the hepatocytes of neonatal pig. In addition, we also explored pGH’s biology in hepatocytes from neonatal pig, it can be found that pGH/GHR could translocate into the cell nucleus, which implies that pGH/GHR may exhibit physiological roles based on their nuclear localization. We found that pGH could not trigger intracellular signaling in the hepatocytes of neonatal pigs, but not young pigs, which provides an important explanation for why the growth of neonatal pig is GH independent.

## Introduction

Growth hormone (GH) plays important roles in the regulation of growth and development in mammals (Lan et al. 2017). GH exerts its physiological functions by binding to growth hormone receptor (GHR) (Brooks and Waters 2010). It is generally believed that GH binding to GHR may induce GHR to produce special conformation change(s). Subsequently, Janus Kinase 2 (JAK2) is activated by tyrosine phosphorylation, which subsequently phosphrylated signal transducer and activator of transcription (STAT) and extracellular regulated protein kinases (ERK1/2) ERK1/2 (Brooks et al. 2014; Waters 2016). These active signaling proteins transport into the cell nuclei, where they regulate gene expression.

It has been demonstrated that porcine growth hormone (pGH) increases growth rate, improves feed efficiency, protein synthesis and increases muscle growth markedly (Chung et al. 1985; Evock et al. 1988). pGH is considered to display its physiological effects through two ways, namely direct effects and indirect effects, the latter is mediated by pGH-induced insulin like growth factor I (IGF-I) (Daughaday and Rotwein 1989). The liver is a major target organ of GH and it is generally believed that the liver is the main source of IGF-I in the circulation under pGH stimulation (Butler and Roith 2001). pGH is the most important hormone that regulates postnatal somatic growth of pig (Wester et al. 1998). However, it is interesting that pGH displaying its bioactivities is closely related to the physiological phases of pig. It has been reported that the growth of neonatal pig is GH independent (Mbler et al. 1992; Harrell et al. 1994). However, some studies have also indicated that neonatal pig is responsive to pGH, but the response level is weaker than that of adult pigs. In addition, although pGH could stimulate the liver of neonatal pig to express IGF-1 mRNA and improve the level of circulating IGF-1, the ability of the production of IGF-1 is weaker than that of young pig (Rehfeldt et al. 2004). Furthermore, the concentration of pGH in the circulation of neonatal pig is very low (Lan et al. 2015), and pGHR expression also can be detectable in many tissues of neonatal pig, such as the liver, muscle and bone (Wester et al. 1998). To date, the reason why pGH is insensitive in neonatal pig remains to be fully understood.

The aim of the present study is (1) to explore intracellular signaling induced by pGH in the hepatocytes of neonatal pig; (2) to find a possible answer for why pGH is not sensitive in neonatal pig from the angle of pGH-induced intracellular signaling. Porcine hepatocyte is an important target cell of pGH and also is an ideal somatic cell model to study pGH-induced intracellular signaling (Lan et al. 2015). Therefore, in the current study, we isolated porcine hepatocytes of neonatal pigs (1–7 days old). We found that pGH could not trigger intracellular signaling in the hepatocytes of neonatal pig, but not young pigs.

## Materials and methods

### Antibody and reagent

Porcine growth hormone and fluorescein isothiocyanate (FITC) were purchased from Sigma (St. Louis, MO, USA). Phospho-JAK2 and JAK2 were from Cell Signaling Technology (Danvers, MA, USA). Phospho-STAT5/3/1 and total STAT5/3/1 antibodies were obtained from Santa Cruz (Santa Fe County, New Mexico, USA). PVDF membranes, ECL and BSA were from Millipore. Porcine GHR, β-actin and normal mouse/rabbit lgG were obtained from Abcam (Cambridge, England). Cell culture plates (6, 12 and 24 well format) were purchased from Corning Costar (Cambridge, MA, USA). Fetal calf serum (FCS) was obtained from Invitrogen (Carlsbad, CA, USA). Lysis buffer was purchased from Beyotime Biotechnology (Shanghai, China). Collagenase was obtained from Hua Cheng Biological Inc (Changchun, China). All other reagents were purchased from Sigma (St. Louis, MO, USA).

### Isolation and culture of porcine hepatocytes

Porcine hepatocytes were isolated according to our previous methods (Lan et al. 2017). In brief, the pigs (Landrace, 1–7 days old and 100 days old) were stunned and exsanguinated. The left liver lobes were cut. A cannula was inserted into the portal vein of the porcine liver. The porcine livers were first perfused with physiological saline buffer to remove out blood cells. The livers were then perfused with the collagenase. Following perfusion, the left lateral lobes of porcine livers were excised and minced. The resulting hepatocyte suspension was filtered through a 100 µm nylon mesh. The porcine hepatocytes were collected by centrifugations at 50 g for 5 min. The resulting hepatocytes were then adjusted to proper concentration and seed into cell culture plates. The study was approved by the Animal Ethical Committee of Jilin Agricultural University. pGH was conjugated to FITC, according to previous protocols (Lan et al. 2015).

### Analysis of pGHR expression on porcine hepatocyte

To evaluate pGHR expression, Western-blot was performed. Freshly isolated hepatocytes were washed three times with ice-cold PBS. The cells were then transferred on ice and lysed in RIPA lysis buffer containing protease inhibitor cocktail. The samples were then collected by centrifugation. The cell lysates were resolved by SDS-PAGE and transferred to PVDF membranes. After blocking with 3% BSA for 60 min, the membranes were incubated with anti-pGHR antibody, control antibody or anti-β-actin antibody for 60 min. After three washes, the membranes were incubated with horseradish peroxidase conjugated secondary antibody. After washing with PBST for three times, the immunoreactive protein bands were detected by using an ECL plus kit.

### Analysis of intracellular signaling in the hepatocytes of neonatal pig

Western-blotting was performed to check the expression level of intracellular signaling proteins, according to our previous methods (Lan et al. 2017). The Western-blot experiments were divided into two parts. First, the expression levels of signaling proteins (namely total proteins) were analyzed. Secondly, the phosphorylation levels of intracellular signaling proteins were evaluated. In brief, the culture media of porcine hepatocytes were replaced with serum-free media for 4 h before the experimental treatments. The porcine hepatocytes were stimulated with pGH for 30 min at 37°C, after which, the porcine hepatocytes were solubilized. The protein samples were then collected, and the protein concentrations were determined using a BCA protein assay kit. The samples were resolved by SDS-PAGE and transferred to PVDF membranes. After blocking with 3% BSA for 60 min, the membranes were incubated with indicated antibodies for 60 min. After washing three times with PBST, the membranes were incubated with horseradish peroxidase (HRP)-conjugated secondary antibody for 60 min. After washing with PBST for three times, the immunoreactive protein bands were detected by using an ECL plus kit. The membranes were incubated with stripping buffer solution for 30 min at 55°C. The membranes were then blocked and re-probed for signaling proteins.

### Laser scanning confocal microscope (CLSM) analysis

Freshly isolated porcine hepatocytes were placed on glass cover slips in 6-well cell culture plates and maintained in serum-free culture media for 2 h. The cells were treated as follows: (1) for pGH internalization analysis, the cells were washed with PBS, the FITC-pGH were then added into the plates and incubated for different durations. The porcine hepatocytes were then washed for three times with PBS and fixed with 4% paraformaldehyde at 37°C for 20 min. After washing, the cell nuclei were stained with DAPI. After washing for three times with PBS, the cells were observed using confocal laser scanning microscopy (Olympus FV3000); (2) for colocalization analysis, the cells were fixed and blocked, the cells were then treated with the indicated antibodies. After washing, the cells were incubated with second antibodies labeled with Alexa Fluor 488 (green) and Alexa Fluor 555 (red). After washing for three times, the cells were observed using CLSM.

### Statistics

The data are presented as the mean ± standard error (S.E.). The results were analyzed by one-way analysis of variance using Statistical Analysis System (SAS) software (SAS version 9.0; Institute Inc., Cary, NC, USA). A *p*-value <0.05 was considered statistically significant.

## Results

### pGHR expression on the hepatocytes of neonatal pigs

**Figure 1.**
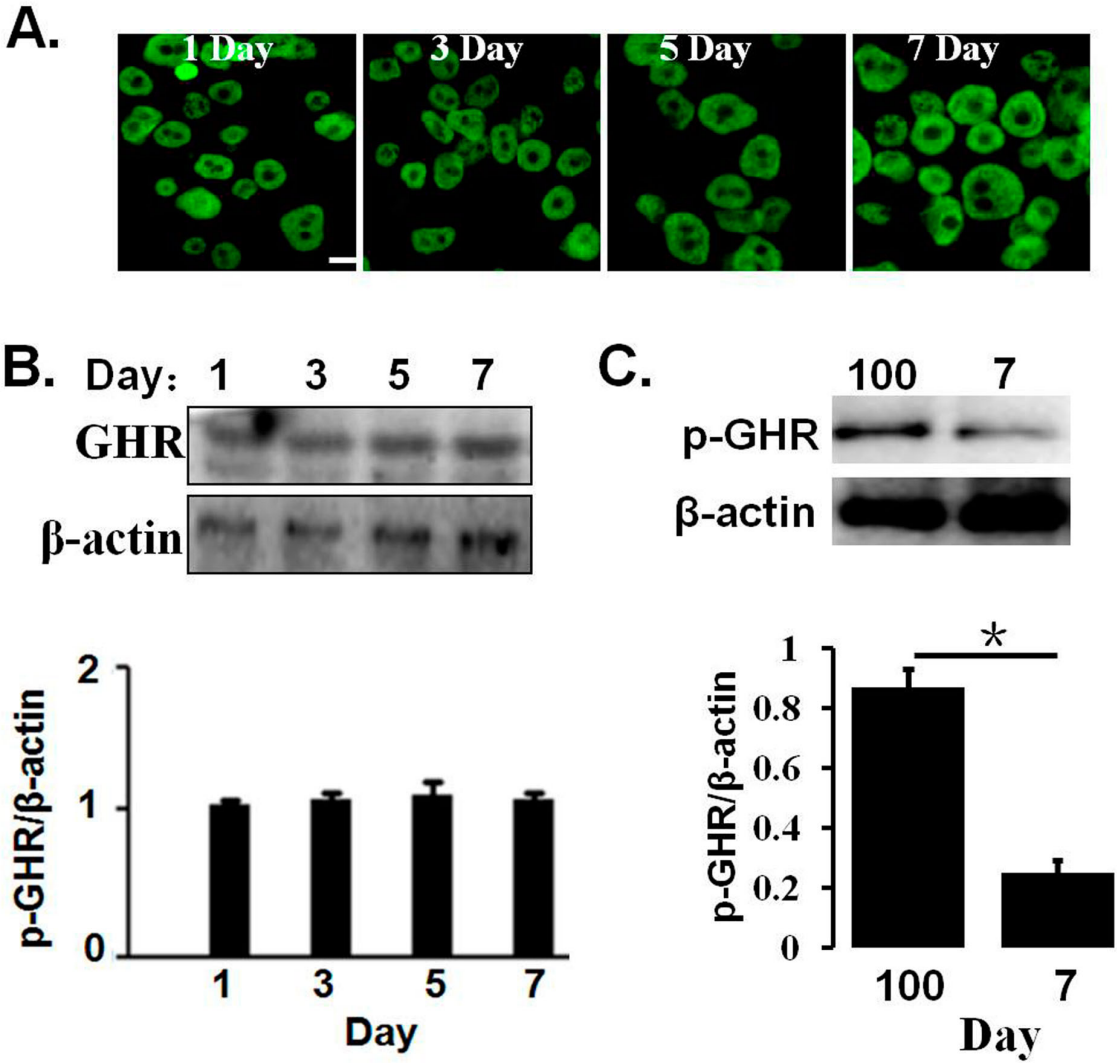
(A) pGHR expression on the hepatocytes from 1 to 7 days old and 100 days old pig. The freshly isolated hepatocytes were pre-treated, as described as in materials and methods. The cells were then incubated with anti-pGHR antibody. After washing three times, the cells were treated with FITC-labeled secondary antibody. Bar: 10 μm. (B) Characterization of pGHR expression by Western blot. The cell extracts from porcine hepatocytes were subjected to immunoblotting with anti-pGHR antibody. After incubation with secondary antibody at room temperature, the immunoreactive bands were detected using an ECL-plus kit. (C) Comparison of pGHR expression between 100 days old pig and 7 days old pig. Data are shown as the mean ± SE. Significant differences are marked with an asterisk. The figure is representative of three independent experiments.

We first preliminarily evaluate hepatic cell surface GHR expression in the hepatocytes of 1–7 days old and 100 days old pig by CLSM, as indicated in Figure 1(A). The results indicated that GHRs were expressed on the cell cytoplasm and membrane, and there were no difference in the pGHR expression levels in 1–7 days old pig. In addition, pGHR expression was also evaluated by Western blotting; the results indicated that the pGHR expression has no change in the hepatocytes of 1–7 days old pig (Figure 1(B)). In addition, pGHR expression from hepatocytes of 100 days old pig is higher than that of 7 days old pig (Figure 1(C)).

### Intracellular signaling proteins expression

**Figure 2.**
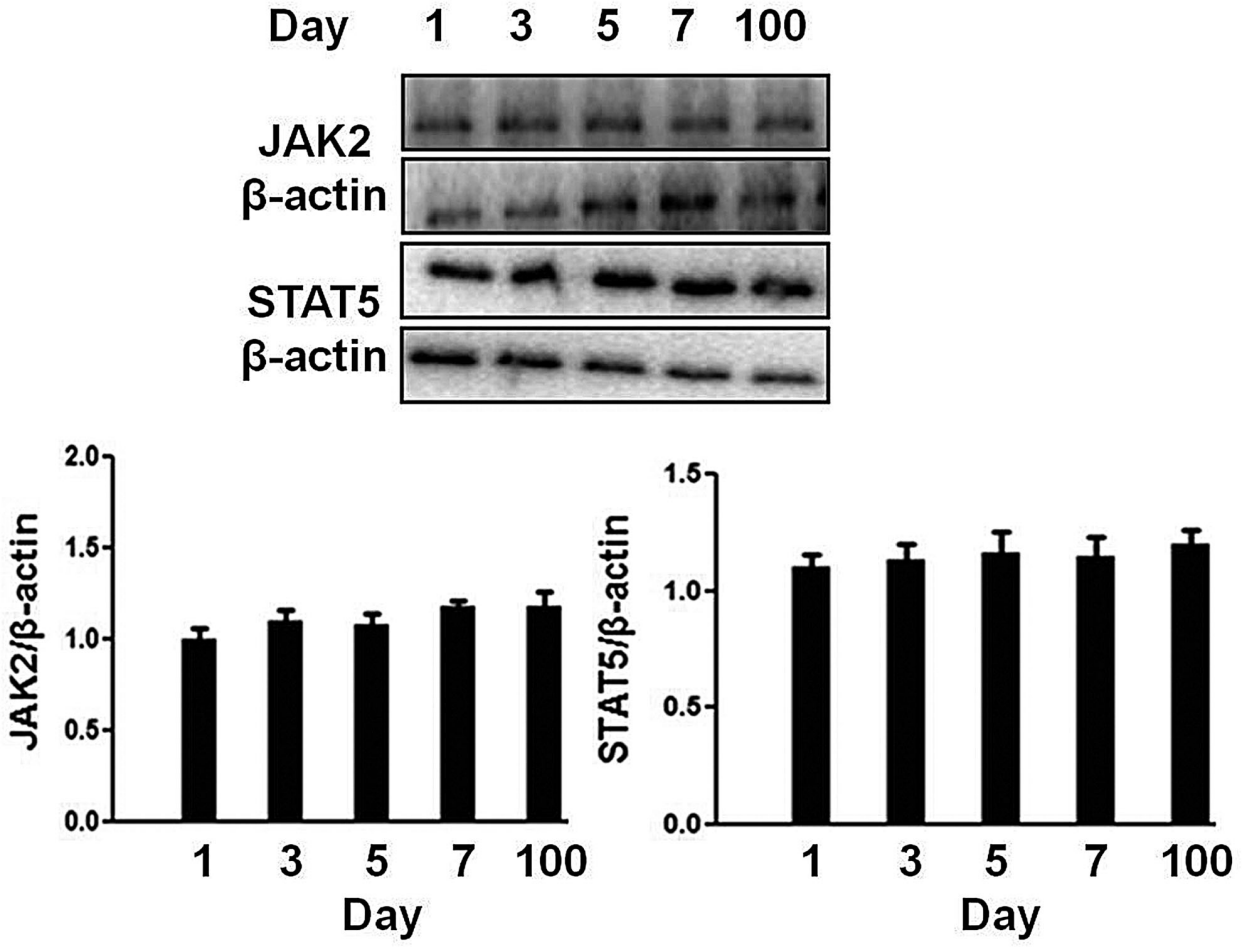
Analysis of signaling protein expression in the hepatocytes obtained from 1 to 7 days old and 100 days old pig. The cell extracts from porcine hepatocytes were subjected to immunoblotting with the indicated antibodies. After incubation with secondary antibody at room temperature, the immunoreactive bands were detected using an ECL-plus kit. The figure is representative of three independent experiments.

We first check the expression level of signaling molecules (JAK2, STAT5) in the hepatocytes of neonatal pigs. As shown in Figure 2, the hepatocytes from 1 to 7 days old pigs expressed a similar level of JAK2 and STAT5 with that of 100 days (∼60 kg). Therefore, porcine hepatocytes from 1, 3, 7 and 100 days pigs were used at the following experiments.

### JAK2 activation induced by pGH in the hepatocytes of neonatal pig

**Figure 3.**
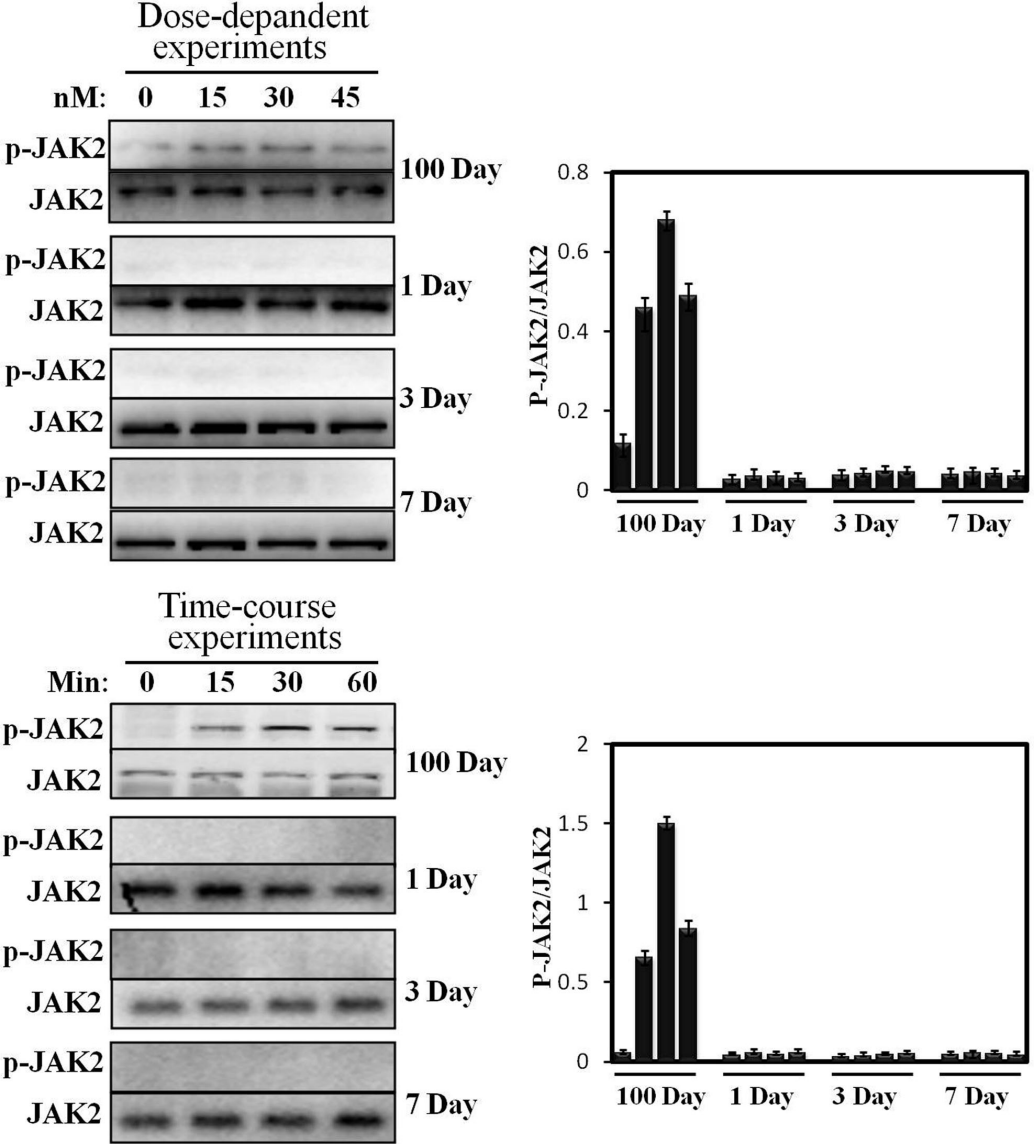
Dose-response and time-course of STAT5 phosphorylation activated by pGH. For dose-response experiments, the cell extracts from porcine hepatocytes obtained from 1 to 7 days old and 100 days old pig were stimulated with increasing concentrations of pGH (0–45 nM) for 30 min, after which, the cell extracts were subjected to immunoblotting with the anti-pJAK2 or anti-total JAK2. After incubation with secondary antibody, the immunoreactive bands were detected using an ECL-plus kit; for time-course experiments, the cell extracts from porcine hepatocytes obtained from 1 to 7 days old and 100 days old pig were treated with constant pGH for different durations at 37°C (0–60 min). After washing for three times, the cellular proteins were solubilized and subjected to immunoblotting with the anti-pJAK2 or anti-total JAK2. The figure is representative of three independent experiments.

Previous studies have reported that the hepatocyte of growing young pig (∼60 kg) is very sensitive to pGH (Lan et al. 2015). Therefore, in the current study, the hepatocytes of 100 days old (∼60 kg) were used as a positive control. In our previous study, JAK2 activation displayed a time-dependent manner (0–60 min). Based on these data, we study JAK2 activation under pGH stimulation in neonatal hepatocytes under the same culture conditions. We can see from Figure 3 that pGH cannot activate JAK2 in time-course experiments in neonatal hepatocytes. In contrast, JAK2 phosphorylation level obviously elevated after pGH stimulation for 15 and 30 min.

### STATs phosphorylation induced by pGH in the hepatocytes of neonatal pig

**Figure 4.**
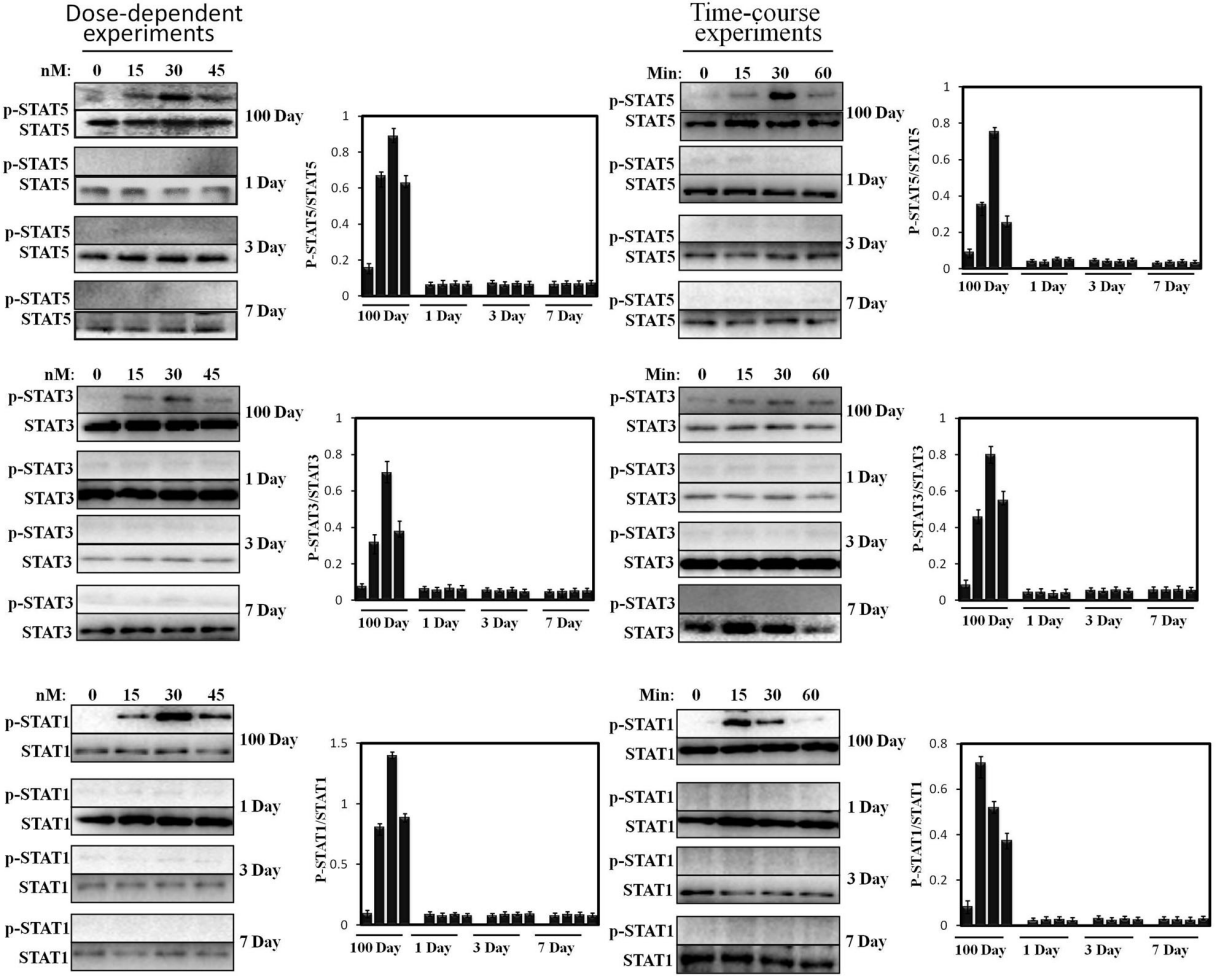
Dose-response and time-course of STAT5/3/1 activation induced by pGH in the hepatocytes obtained from 1 to 7 days old and 100 days old pig. For dose-response experiments, the cell extracts from porcine hepatocytes obtained from 1 to 7 days old and 100 days old pig were stimulated with increasing concentrations of pGH (0–45 nM) for 30 min, after which the cell extracts were subjected to immunoblotting with the anti-pSTAT5/3/1 or anti-total STAT5/3/1. After incubation with secondary antibody for 1 h, the immunoreactive bands were detected using an ECL-plus kit; for time-course experiments, the cell extracts from porcine hepatocytes obtained from 1 to 7 days old and 100 days old pig were treated with constant pGH for different durations at 37°C (0–60 min). After washing for three times, the cellular proteins were solubilized and subjected to immunoblotting with the anti-pSTAT5/3/1 or anti-total STAT5/3/1. The figure is representative of three independent experiments.

Subsequently, STAT5/3/1 activation was accessed. Similar to JAK2, our previous experimental and others have reported that the hepatocytes of growing young pig (60 kg) are very sensitive to pGH, which exhibits sensitive STATs’ signaling response in time-course experiments. Based on this, we investigated STAT5/3/1 activation under pGH stimulation in neonatal hepatocytes under the same culture conditions. As shown in Figure 4, pGH cannot induce STAT5/3/1 phosphorylation in dose- and time-dependent experiments in neonatal hepatocytes. In contrast to neonatal hepatocytes, STAT5/3/1 was strongly activated under pGH stimulation in a dose- and time-dependent manner.

### Exploration of possible mechanisms for JAK2-STATs’ signaling insensitivity

**Figure 5.**
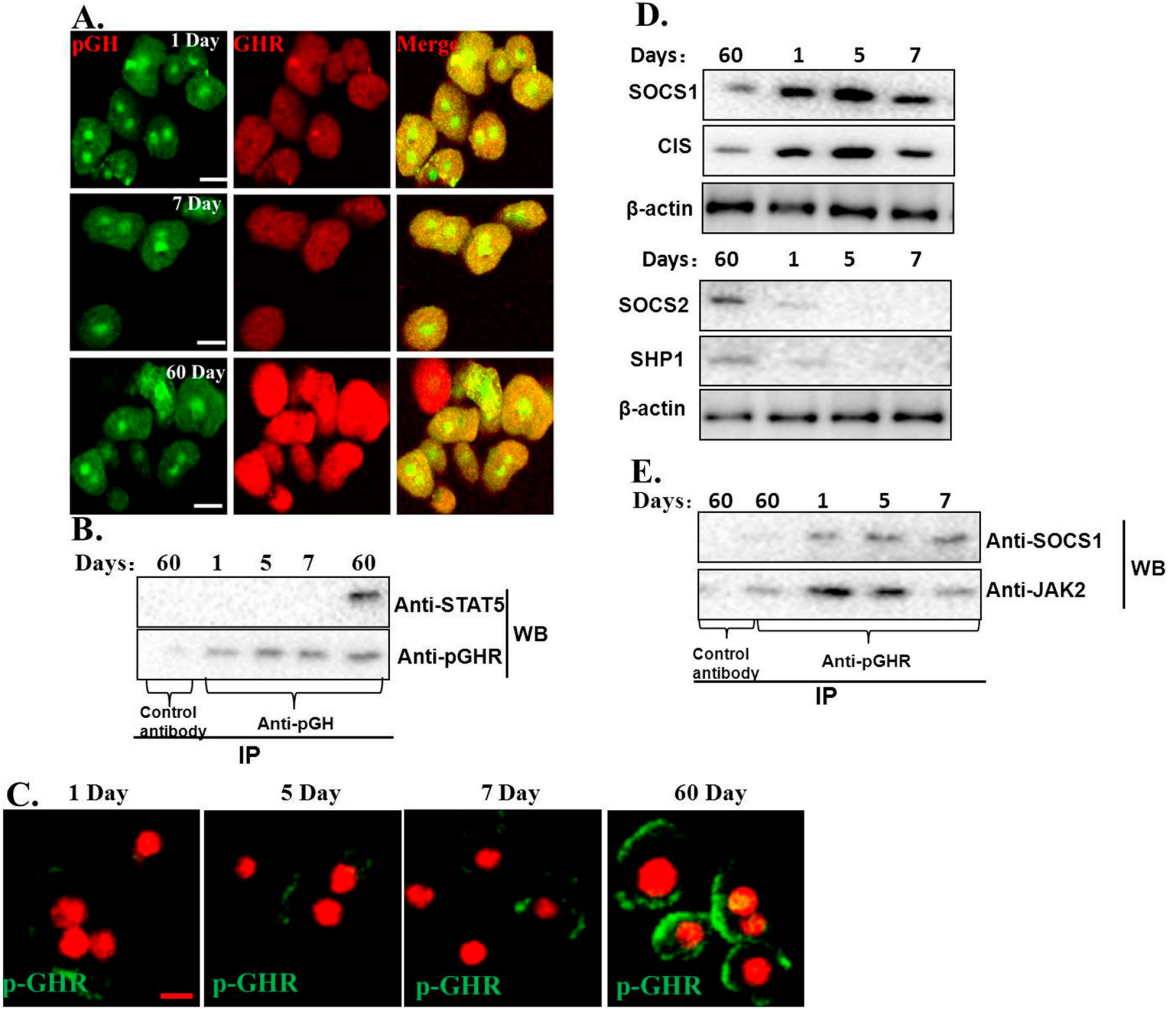
Exploration of possible mechanisms for JAK2-STAT5 signaling insensitivity. A. pGH could interact normally with GHR in the hepatocytes of neonatal pig. The cells were then washed for three times with PBS and fixed with 4% paraformaldehyde at 37°C for 20 min. The cells were then treated with the indicated antibodies. After washing, the cells were incubated with second antibodies labeled with Alexa Fluor 488 (green) and Alexa Fluor 555 (red). The cells were observed using confocal laser scanning microscopy (CLSM). Bar: 10 μm. B. pGH/GHR lacks the ability to recruit and activate intracellular signaling molecule. C. The plasma membrane-localized GHR was not activated by pGH in hepatocytes of neonatal pig. D. The expression of negative regulatory molecules for JAK2-STAT5 signaling. E. SOCS1 could interact with JAK2/GHR complex in the hepatocytes of neonatal pig.

We first checked the interactions between pGH and GHR by CLSM; we can see that pGH and GHR could interact with each other on the cells, which indicated that pGH could interact normally with GHR (Figure 5(A)).

The above-mentioned study has shown that pGH could interact normally with GHR. We then analyzed if pGH/GHR could recruit intracellular signaling molecules in hepatocytes of neonatal pig, the results showed that pGH/GHR lacks the ability to recruit and activate intracellular signaling protein (STAT5) (Figure 5(B)). Next, we ask why pGH/GHR cannot recruit and trigger the intracellular signaling protein. It is well known that GHR phosphorylation is necessary for STAT5 activation; we, therefore, tested whether GHR was phosphorylated after pGH treatment, the results indicated that the plasma membrane-localized GHR was not phosphorylated in hepatocytes of neonatal pig in our experimental conditions (Figure 5(C)).

Next, we further explore why GHR was not activated by pGH in the hepatocytes of neonatal pig. Previous studies have demonstrated that JAK2-STAT5 signaling pathway is regulated by negative regulatory molecules (such as tyrosine-phosphatases SHP-1,-2 and suppressors of cytokine signaling (CIS/SOCS)). Therefore, we checked the expression of these negative regulatory molecules, as shown in Figure 5(D), SOCS1 was highly expressed. To further test whether this negative regulatory molecule is involved in pGH/GHR signaling insensitivity in the hepatocytes of neonatal pig, we performed IP and WB experiments, and the results indicated that SOCS1 could interact with JAK2/GHR complex in the hepatocytes of neonatal pig (Figure 5(E)). These observations suggest that SOCS1 could play a role in the pGH/GHR signaling insensitivity in the hepatocytes of neonatal pig.

**Figure 6.**
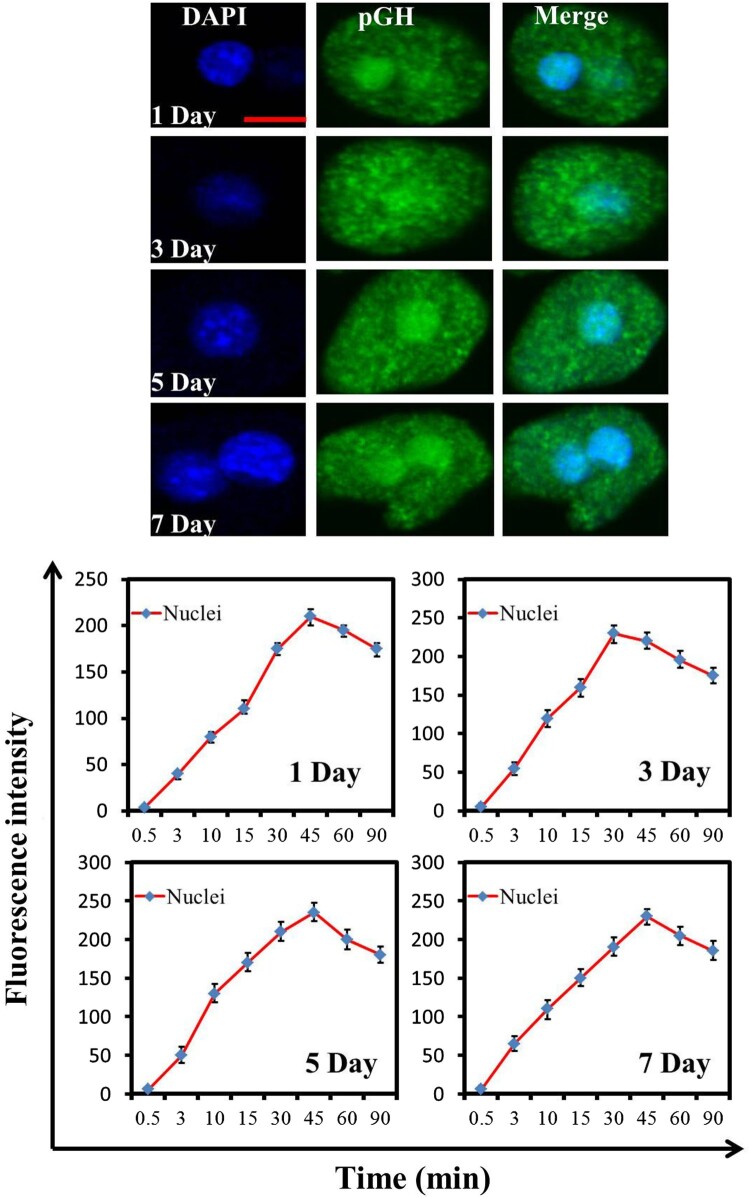
pGH’s nuclear localization in the hepatocytes of neonatal pig. Freshly isolated porcine hepatocytes were placed on glass coverslips. After incubation with serum-free culture media for 3 h, the FITC-pGH were added into the plates and incubated for different durations. The cells were observed using confocal laser scanning microscopy (CLSM). Bar: 10 μm. The fluorescence intensity at the cell nuclei was determined by selecting the appropriate region of interest (ROI) using image J software (developed by NIH). The numeric data are presented as mean ± SD from at least three independent experiments with 30 cells each.

Since pGH/GHR fails to activate the intracellular signaling pathway in the hepatocytes of neonatal pig, does it mean that pGH/GHR has no effect in neonatal pig? Traditional view believes that pGH/GHR exhibits its physiological functions in cell membrane; however, recent studies also show that GH/GHR can transport into cell nuclei, where they also can exert important roles (such as proliferation) (Conway-Campbell et al. 2007). Therefore, GHR’s nuclear localization induced by GH should be a potential parameter for evaluating pGH/GHR’s activity except for intracellular signaling. Here, we preliminarily investigated pGH’s nuclear translocation in the hepatocytes of neonatal pig. As shown in Figure 6, pGH could translocate into cell nuclei of hepatocytes of neonatal pig, which suggests that, in this physiological stage (namely newborn pigs), pGH could exhibit its functions based on its nuclear localization in hepatocytes of neonatal pig.

## Discussion

Although pGH is one of the most important hormones that regulates porcine postnatal growth and development, many studies indicated that neonatal pig is irresponsive or insensitive to pGH in neonatal pig (Harrell et al. 1994; Rehfeldt et al. 2004). However, until now, the reason for this phenomenon remains to be fully understood. In this study, we try to investigate this problem from the angle of intracellular signaling induced by pGH. To our knowledge, this study initially investigates pGH-induced intracellular signaling pathway in the hepatocytes of neonatal pig.

In previous studies, the researchers have analyzed the reason for why GH is not sensitive in neonates; they proposed that GHR’s expression is low in neonatal animals, which leads to low GH binding, which, in turn, results in low circulating IGF-1 (Martinez et al. 2013). However, Wester et al reported that they investigated the effects of exogenous GH treatment on IGF-I level in the circulation and found that IGF-I in the plasma is increased in vivo (Wester et al. 1998). In addition, Lewis et al indicated that exogenous GH administration results in increasing IGF-1 and GHR mRNA expression. These results suggest that GH is responsive in neonatal pigs. However, the magnitude of the responses is considerably lower than that of mature pigs (Lewis et al. 2000). In the current study, we try to find a new explanation from the angle of pGH-induced intracellular signaling. We found that pGH cannot activate JAK2-STATs’ signaling in the hepatocytes of newborn pigs under our experimental conditions (Figures 3 and 4). However, pGH could strongly activate JAK2-STATs in porcine hepatocytes from 60 kg pigs. This work suggests that weak or no signaling responsiveness may result in that pGH is not sensitive in newborn pigs. Although Lewis’s research has shown that, in newborn pigs, IGF-I mRNA expression is weakly elevated in the liver and muscle under exogenous GH treatment, muscle may be more responsive to GH than the liver in this physiological stage (namely newborn pigs) (Lewis et al. 2000), which suggests that, in newborn pigs, IGF-1 in the circulation was mainly derived from muscle or other organ, but not liver. This suggests that the main source of IGF-1 is different in different physiological stages; this implies a complementary mechanism among different tissues may exist, by which the level of plasma IGF-1 maintains a normal physiological status.

GH is considered to display its physiological effects through two ways, direct effects and indirect effects, the latter is mediated by the insulin like growth factor I (IGF-I) (Sjögren et al. 1999). The liver is believed to be the primary source of circulating IGF-I (Le et al. 2001). The IGF-I gene expression is regulated by JAK2-STAT5 signaling pathway. In this work, JAK2-STAT5 signaling is undetectable in the hepatocytes from newborn pigs, which provide a possible explanation for low IGF-1 expression in newborn pigs.

Recently, a series of studies have shown that pGH not only exhibits its physiological roles in cell membrane but also exerts its biological activities by its nuclear translocation (Brooks et al. 2008). It has been shown that the nuclear-localized GHR exhibits a strong relation with cell’s high proliferation (Conway-Campbell et al. 2007). In the current study, it can be observed that pGH was rapidly translocated into the nucleus by CLSM, which suggests that pGH/GHR may directly participate in cell proliferation. Based on these, we propose a new paradigm for pGH/GHR’s roles, namely pGH may be mainly involved in liver cell proliferation but not in IGF-1 expression in neonatal pig.

In summary, in the current work, we found that JAK2-STATs signaling is not sensitive to pGH in newborn pigs, which provides a possible explanation for why pGH is unresponsive in newborn pigs. Based on this, we propose a new paradigm for pGH’s functions in neonatal pig.
